# Identification of telomere dysfunction in Friedreich ataxia

**DOI:** 10.1186/s13024-015-0019-6

**Published:** 2015-06-10

**Authors:** Sara Anjomani Virmouni, Sahar Al-Mahdawi, Chiranjeevi Sandi, Hemad Yasaei, Paola Giunti, Predrag Slijepcevic, Mark A. Pook

**Affiliations:** Division of Biosciences, Department of Life Sciences, College of Health & Life Sciences, Brunel University London, Uxbridge, UB8 3PH UK; Synthetic Biology Theme, Institute of Environment, Health & Societies, Brunel University London, Uxbridge, UK; Current address: Uro-Oncology Research Group, Cancer Research UK-Cambridge Institute, University of Cambridge, Cambridge, UK; Department of Molecular Neuroscience, Institute of Neurology, University College London, Queen Square, London, UK

**Keywords:** Friedreich ataxia, FRDA, frataxin, *FXN*, GAA repeat expansion, Mouse model, Alternative lengthening of telomeres, ALT, Telomere dysfunction

## Abstract

**Background:**

Friedreich ataxia (FRDA) is a progressive inherited neurodegenerative disorder caused by mutation of the *FXN* gene, resulting in decreased frataxin expression, mitochondrial dysfunction and oxidative stress. A recent study has identified shorter telomeres in FRDA patient leukocytes as a possible disease biomarker.

**Results:**

Here we aimed to investigate both telomere structure and function in FRDA cells. Our results confirmed telomere shortening in FRDA patient leukocytes and identified similar telomere shortening in FRDA patient autopsy cerebellar tissues. However, FRDA fibroblasts showed significantly longer telomeres at early passage, occurring in the absence of telomerase activity, but with activation of an alternative lengthening of telomeres (ALT)-like mechanism. These cells also showed accelerated telomere shortening as population doubling increases. Furthermore, telomere dysfunction-induced foci (TIF) analysis revealed that FRDA fibroblasts have dysfunctional telomeres.

**Conclusions:**

Our finding of dysfunctional telomeres in FRDA cells provides further insight into FRDA molecular disease mechanisms, which may have implications for future FRDA therapy.

**Electronic supplementary material:**

The online version of this article (doi:10.1186/s13024-015-0019-6) contains supplementary material, which is available to authorized users.

## Background

Friedreich ataxia (FRDA) is an autosomal recessive neurodegenerative disorder caused by GAA repeat expansion mutation within intron 1 of the *FXN* gene. This leads to reduced frataxin expression, defective iron-sulphur cluster (ISC) formation, mitochondrial iron accumulation and oxidative stress, with eventual neuronal cell death. Previous studies have reported FRDA fibroblasts to be more sensitive to ionising radiation than control cells, suggesting that FRDA may be a DNA damage response-deficient disorder [1]. This is supported by gene expression studies of human peripheral blood leukocytes that have indicated involvement of DNA repair pathways in FRDA [2, 3]. It has also been well documented that oxidative damage to DNA and defects of DNA damage responses can cause accelerated rates of telomere attrition and chromosomal instability [4]. Furthermore, a recent study of human peripheral blood leukocytes has indicated telomere shortening in FRDA patients compared to healthy controls [5]. Therefore, we aimed to further investigate telomere maintenance in FRDA cells.

Telomeres play an essential role in the maintenance of genomic stability via chromosome-end protection [6]. These specialised nucleoprotein structures form a loop to protect the chromosome ends from exonuclease degradation and terminal fusions. Degradation of telomeres can be caused by unresolved end-replication, exonuclease activity or DNA breakage within telomeric sequences due to oxidative damage [4, 7, 8]. Telomere length maintenance is carried out either by the activity of a telomere-specific DNA polymerase called telomerase or by a telomerase-independent pathway referred to as alternative lengthening of telomeres (ALT) [6]. ALT cells are characterised by recombinational events at telomeres, known as telomeric sister chromatid exchanges (T-SCE), and co-localisation of telomeres and promyelocytic leukemia protein (PML) nuclear bodies [9]. It is thought that ALT-associated PML bodies (APBs) could provide templates for replication and recombination-based telomere lengthening to enhance telomere elongation or it may aid in recruitment of proteins to the telomeric regions to facilitate inter-telomeric recombination [10]. Normal human somatic cells do not have telomerase or ALT activity, thus after a limited number of divisions the cell population undergoes telomere-mediated senescence due to short dysfunctional telomeres [11]. However, immortalised human cell lines either activate telomerase or engage the ALT mechanism to maintain telomeres through recombination. Therefore, the telomere length is generally stable in these cells since equilibrium exists between telomere degradation and telomere renewal [6].

Here, we have analysed the telomere length and rate of telomere shortening in FRDA human and transgenic mouse fibroblasts. We report that there is an initial comparative increase of telomere length in FRDA cells due to ALT-like activation, followed by an increased rate of telomere attrition due to telomere dysfunction, which may be caused by a combination of oxidative stress and defective DNA repair mechanisms. We also confirmed the previous report of reduced telomere length in FRDA peripheral blood leukocytes [5].

## Results

### Telomere length analysis in human and mouse FRDA cells and tissues

**Fig. 1:**
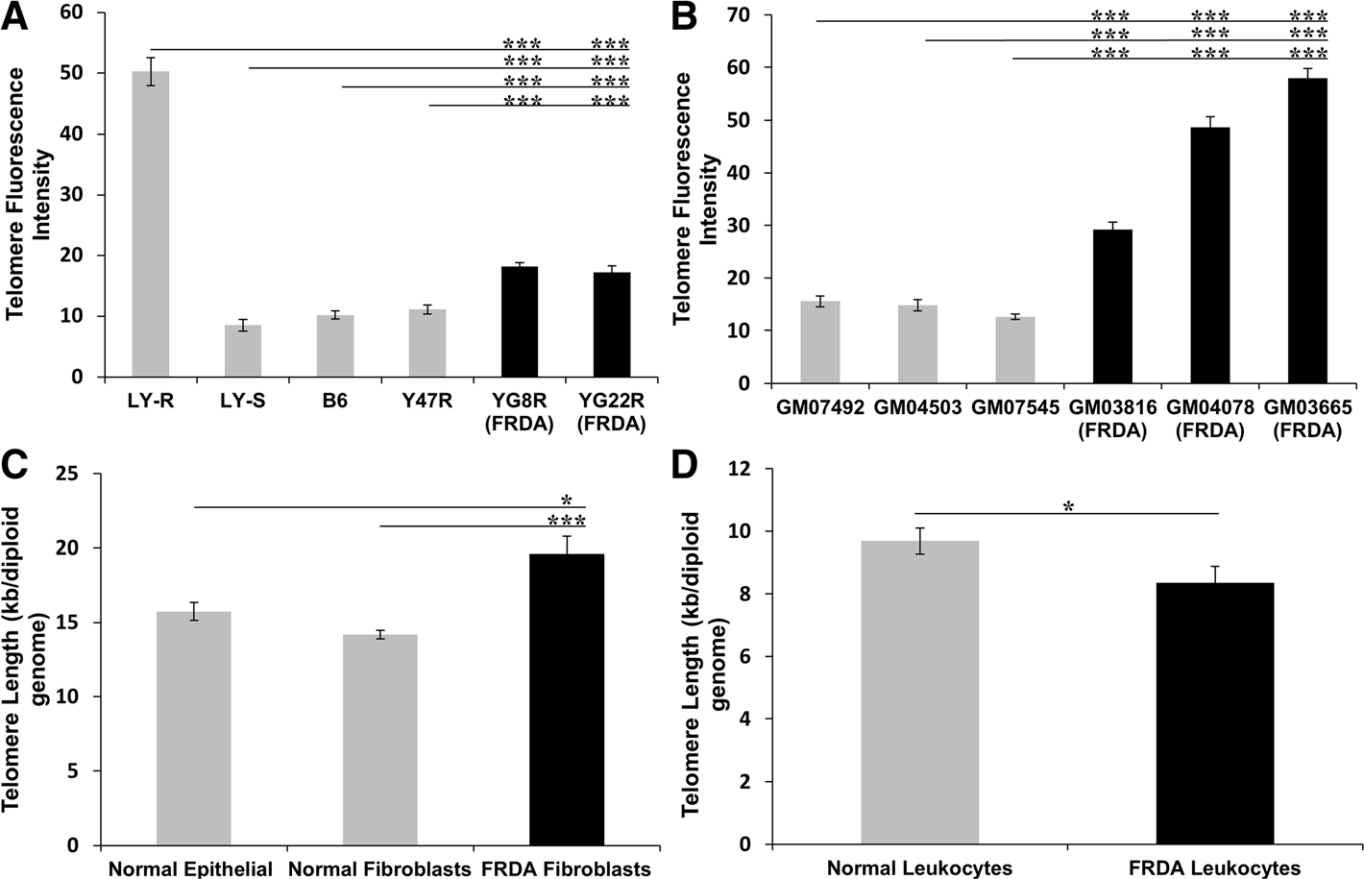
Telomere length analysis of FRDA cells. **a** Mean telomere length determinations of mouse FRDA fibroblast cells by Q-FISH, showing significantly increased telomeric repeat fluorescence in YG8R and YG22R FRDA mice compared to Y47R and B6 controls. The LY-R long telomere control also showed 6 times greater telomere fluorescence than the LY-S short telomere control. *n* = 100. **b** Mean telomere length of human FRDA fibroblasts by Q-FISH showed significantly higher telomere fluorescence in all the FRDA fibroblast cells compared to the controls. *n* = 150. **c** Mean telomere length of the FRDA patient fibroblasts (*n* = 3) measured by qPCR was significantly greater than the normal fibroblast (*n* = 3) and epithelial controls (*n* = 3). **d** Mean telomere length of the FRDA leukocytes (*n* = 18) measured by qPCR was significantly lower compared to the controls (*n* = 12). All data are shown as mean ± SEM (^*^ *P* < 0.05 and ^***^ *P* < 0.001, evaluated by Student’s *t* test). All qPCR runs were performed in triplicate and each experiment was repeated independently, at least twice

The telomere length in FRDA human and transgenic mouse fibroblasts was measured by a Q-FISH protocol adapted for interphase cells. A total of 100–150 interphase nuclei per cell line were captured and the mean telomere fluorescence intensity per cell was used to determine the mean difference between FRDA fibroblasts and controls. Initially, telomere fluorescence intensity was analysed in mouse FRDA (YG8R and YG22R) and control (Y47R and B6) fibroblasts at passage 7. To quantify the results, two mouse lymphoma cell lines, LY-R and LY-S, with known telomere lengths of 49 kb and 7 kb, respectively, were used as calibration standards [12]. The results revealed that the mouse FRDA cell lines, YG8R and YG22R, have significantly increased telomeric repeat fluorescence (*P* < 0.001) compared to the controls, Y47R and B6 (Fig. 1a). LY-R control cells were shown to have approximately 6 times greater telomere fluorescence than LY-S control, which confirmed the accuracy of the technique. Subsequently, telomere fluorescence intensities of PNA probes were analysed in human FRDA and control fibroblasts that had each undergone 7 passages subsequent to acquisition from the Coriell Cell Repository (details are shown in Table 1). The results showed that the mean telomere fluorescence intensity of the PNA probes, and hence telomere length, in all FRDA fibroblast cells was significantly greater (*P* < 0.001) than those of controls (Fig. 1b). The values for human FRDA cells also appeared to be higher than those of the FRDA mouse cells, suggesting potential differences due to factors such as GAA repeat size. However, a direct comparison between these human and mouse results cannot effectively be made due to the relative rather than absolute nature of the two experiments. To further confirm the Q-FISH results, qPCR was performed to evaluate the telomere length in the human FRDA and control cell lines. Three epithelial cell samples with known telomere length, previously measured by telomere repeat amplification (TRF), were used as the controls to examine the accuracy of the technique. The results obtained by qPCR were in good agreement with those previously acquired by Q-FISH and TRF methods. Overall, the results demonstrated that FRDA fibroblasts have significantly higher average telomere length of 19 kb compared to normal fibroblasts (14 kb, *P* < 0.001) and normal human breast epithelial cells (15 kb, *P* < 0.05) (Fig. 1c).

**Table 1:** Details of the human primary fibroblast cell lines

Samples	Sex	Age (Year)	Race	Number of GAA repeats	Starting passage number (Coriell)
GM08399	Female	19	N/A	Normal	4
GM04503	Female	31	Caucasian	Normal	12
GM07492	Male	17	Caucasian	Normal	3
GM07545	Female	22	Caucasian	Normal	5
GM03816	Female	36	Caucasian	330/380	5
GM04078	Male	30	Caucasian	541/420	2
GM03665	Female	13	Caucasian	445/740	7

Subsequently, peripheral blood leukocyte genomic DNA samples from 18 FRDA patients and 12 healthy controls (details shown in Table 2) were used to measure the telomere length by qPCR. The results indicated that, although there was a considerable variation in the telomere length of the patients (Additional file 1), the mean telomere length was significantly (*P* < 0.05) shortened in FRDA leukocyte cells compared to controls (Fig. 1d). These results are in good agreement with those previously reported by Castaldo and colleagues [5]. Although we were not able to investigate any correlation between telomere length and age, as this data was not available to us, we did investigate the correlations between telomere length and gender or GAA repeat sizes, but no significant correlations were observed (Additional file 2).

**Table 2:** FRDA patient leukocyte DNA samples

Samples^a^	Sex	Number of GAA repeats
CA	Male	750/550
MH	Male	1100/450
HK	Male	750/630
LK	Male	730/600
CR	Male	930/570
RS	Female	930/600
MS	Female	700/700
JT	Male	780/780
AB	Female	950/300
AG	Female	900/550
BL	Male	1060/730
BQ	Female	950/250
BT	Male	930/760
IC	Female	950/950
LH	Female	800/250
MB	Female	1000/250
RB	Male	850/750
SK	Female	900/650

^a^NB: Patient ages were not available

Next, we measured the telomere lengths of DNA samples from cerebellum autopsy tissues from FRDA patients compared with age-matched unaffected individuals to provide *in vivo* validation of our observation made from fibroblast cells *in vitro*. Human DNA sample details are shown in Additional file 3. The results showed that telomere lengths in FRDA cerebellar tissues were significantly reduced (*P* < 0.05) compared to those of unaffected age-matched controls. However, there were no correlations between telomere length and age of onset or GAA allele sizes (Additional file 4).

### Telomere elongation in FRDA fibroblasts is due to an ALT-like mechanism and not telomerase activity

**Fig. 2:**
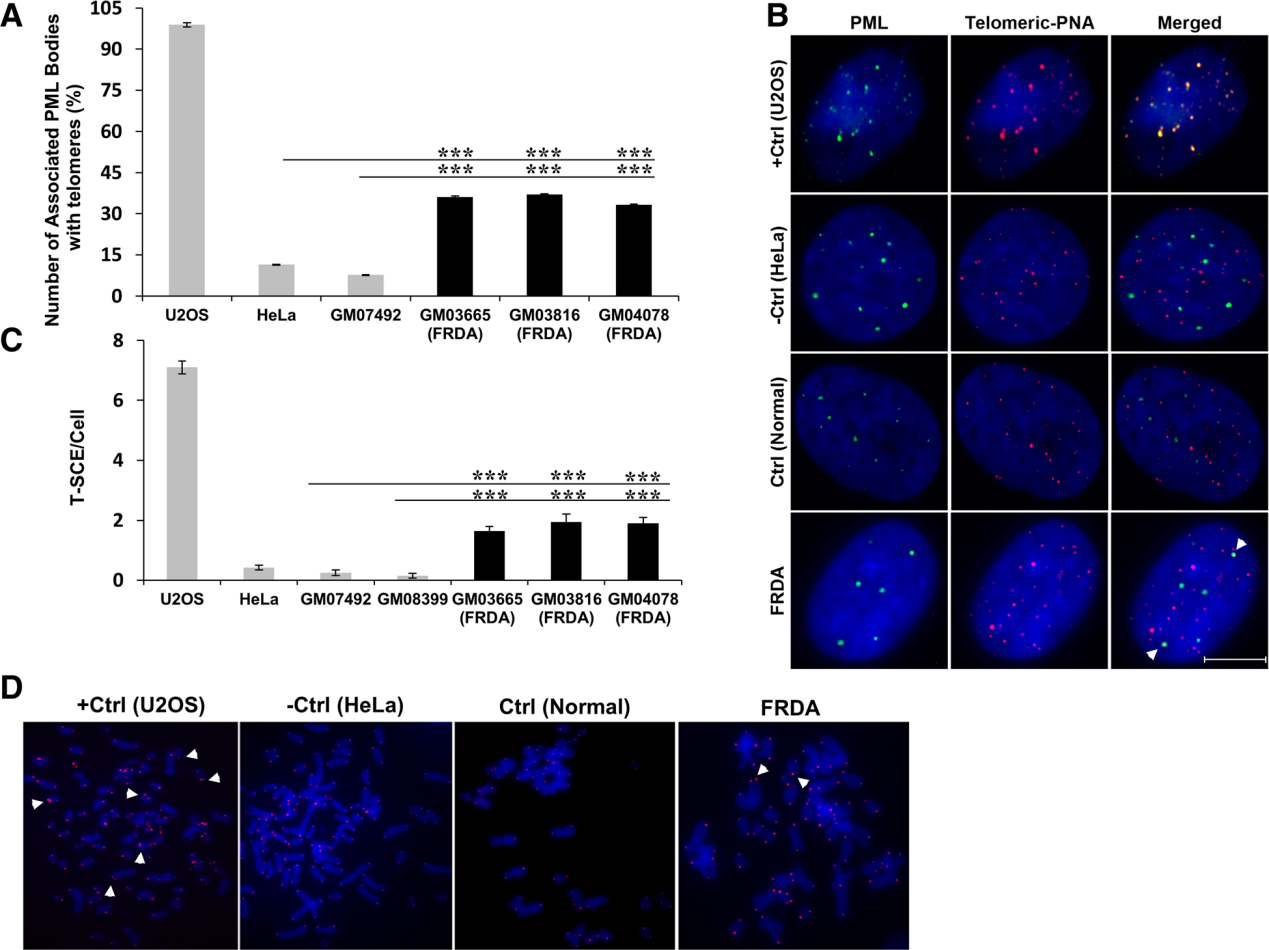
ALT activation in FRDA fibroblasts. **a** Percentage of the PML bodies associated with telomeres demonstrate high co-localisation in all FRDA cells, GM03665, GM03816 and GM04078, compared to GM07492 and HeLa controls. 99 % of the telomere foci co-localised with the PML bodies in U2OS ALT-positive control cells, *n* = 50. **b** Representation of APBs detected by immuno-FISH in FRDA and control (normal) fibroblast cells, U2OS ALT-positive control cells (+Ctrl) and HeLa ALT-negative control cells (−Ctrl). Interphase cells were stained with anti-PML (green) and telomeric Cy3-labeled PNA probes (red). Nuclei were counterstained with DAPI. Scale bar = 10 μm. White arrowhead indicates an APB in a FRDA cell. **c** T-SCE analysis of human FRDA cells by CO-FISH showed a significant increase (*P* < 0.001) in T-SCE levels of FRDA cells compared to the controls, *n* = 20–50. **d** Representative CO-FISH results, showing T-SCEs events (indicated by arrowheads) in FRDA and control (normal) fibroblasts, U2OS ALT-positive controls (+Ctrl) and HeLa ALT-negative controls (−Ctrl). Graph data are shown as mean ± SEM (^***^ *P* < 0.001, evaluated by Student’s *t* test). All the experiments were repeated independently, at least twice

As FRDA fibroblasts were found to have chromosomes with relatively longer telomeric repeats, it was hypothesized that these cells might have overcome telomere shortening through activation of telomerase. To investigate this hypothesis, human FRDA fibroblast cells were screened for expression of telomerase activity using the telomeric repeat amplification protocol (TRAP) assay. In this method, telomerase adds telomeric repeats to the forward primer, provided that it is active in these cells. The extended primer would then be amplified by qPCR with a reverse primer complementary to the telomeric repeat. The results revealed that telomerase activity was not present in FRDA fibroblasts (Additional file 5). Consequently, the FRDA cells were considered to be negative for telomerase activity. Since the TRAP assay is a semi-quantitative method and there exists a strong correlation between human telomerase reverse transcriptase (hTERT) mRNA expression levels and telomerase activity [13], hTERT mRNA expression was measured in FRDA cells. The quantitative assay for hTERT mRNA expression was conducted using TaqMan qRT-PCR. The results indicated that FRDA cells do not express the *hTERT* gene, further confirming lack of telomerase activity in these cells (Additional file 5). Since FRDA fibroblasts exhibited longer telomeres with no detectable telomerase activity, we investigated another mechanism of telomere length maintenance, commonly known as ALT, by measuring ALT-associated PML bodies (APBs). The enhanced formation of APBs in cells is an indicator of ALT activity. FRDA cells were first examined for the presence of PML bodies using immunofluorescence. No difference was detected in the number and distribution of PML nuclear foci in FRDA cells compared to controls. Subsequently, co-localisation of PML bodies with telomeres was investigated in these cells using combined immunofluorescence to PML followed by Q-FISH for telomere detection (Fig. 2a,b). The results indicated that in 50 randomly analysed FRDA cells, approximately 35 % of the PML foci co-localised with telomeric DNA. By comparison, the mean co-localisation values of PML and telomeres in ALT positive U2OS cells, normal human fibroblasts and HeLa negative controls were 99 %, 11 % and 8 % respectively (Fig. 2a,b). These results suggested that an ALT-like mechanism is involved in FRDA cells but it is not as prominent as in ALT positive cells. Previous results have shown that cellular stress may promote aggregation of PML bodies or conversely disperse them into microspeckles [14]. Therefore, the size of co-localised PML bodies with telomeres was examined. However, no significant difference was detected in the size of telomere-associated PML bodies compared with non-telomere-associated PML bodies in FRDA cells (Additional file 6). Consequently, these results did not provide any further information regarding the assembly or disassembly of PML bodies in FRDA cells due to cellular stress. Subsequently, telomere recombination frequencies between sister telomeres were analysed in human FRDA and control primary fibroblasts, using the chromosome orientation FISH (CO-FISH) method. This method is used to measure levels of T-SCE – another marker of ALT activity in cells. The results indicated a significant increase (*P* < 0.001) in T-SCE levels in FRDA cells compared to controls (Fig. 2c,d). These results suggested that T-SCEs occur much more frequently in FRDA cells than normal cells, further confirming the existence of an ALT-like mechanism in these cells.

### Accelerated telomere shortening in FRDA fibroblasts

**Fig. 3:**
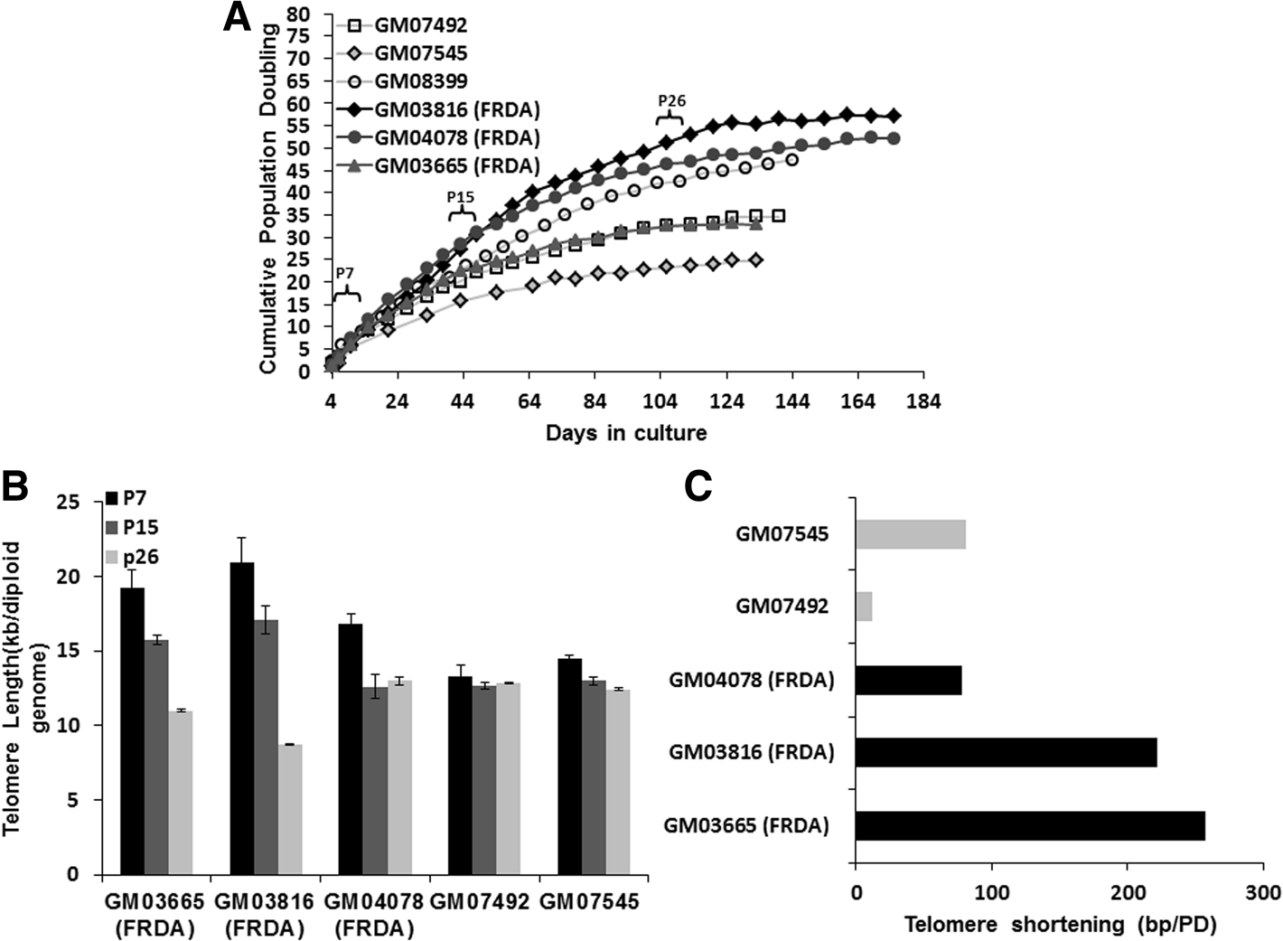
Increase of time to senescence and accelerated telomere shortening of FRDA fibroblasts. **a** Cumulative population doubling (CPD) times of human FRDA and normal fibroblast cell lines. Every cell passage is indicated by a point and the CPD was calculated based on the sum of the cell proliferations over the cumulative time taken from the earliest passages until cells underwent senescence. **b** The average rates of telomere shortening in the human FRDA and normal fibroblasts in bp/population doubling. P7, P15 and P26 = 7th, 15th and 26th passages following acquisition from the Coriell Cell Repository (Table 1), respectively. Data are shown as mean ± SEM. All the experiments were repeated at least twice independently, *n* = 3. **c** The telomere length shortening (bp/PD) of FRDA fibroblasts compared to controls throughout growth

To investigate whether FRDA fibroblasts can activate an ALT-like mechanism during culturing *in vitro* to become immortalised, growth curves and cumulative population doubling time analyses were performed. Growth curve analysis of human FRDA and control fibroblasts was carried out from the earliest passage number available for each cell line. FRDA fibroblasts underwent growth arrest after 124 to 175 days with average cumulative population doubling of 47. In contrast, normal fibroblasts exhibited earlier growth arrest, after 124 to 138 days, with an average of 36 cumulative population doubling (Fig. 3a). These results indicated that FRDA cells were not immortalised by the ALT-like mechanism alone, suggesting that the frequency of the PML bodies may not have been sufficient to prevent senescence. However, FRDA cells senesced on average approximately 11 population doublings later than the controls (*P* < 0.001). The presence of senescent cells was confirmed by β-galactosidase (β-Gal) assay (Additional file 7). This delay in transition from proliferation to senescence in FRDA cells could in part be due to the difference in the original telomere lengths of these cell lines. Subsequently, the effect of replicative aging of culture cells at the 7th, 15th and 26th passages following acquisition from the Coriell Cell Repository (Table 1) was studied by qPCR telomere length assay to determine telomeric DNA loss due to cell divisions. The results of this analysis revealed an accelerated telomere shortening in FRDA fibroblasts, taking initial differences in passage numbers into consideration (Fig. 3b). Although initial results indicated that FRDA fibroblasts contained relatively longer telomeres compared to controls after 7 passages, further analysis after 15 and 26 passages showed that these cells reduced telomere length more rapidly throughout culture than normal cells (Fig. 3b). FRDA fibroblasts showed an average telomere loss of 186 bp per population doubling (PD), which was four times greater than the 47 bp average value of normal fibroblasts (Fig. 3c). The accelerated telomere shortening observed in FRDA fibroblasts suggested that the telomeres may be dysfunctional in these cells.

### Investigation of telomere dysfunction in FRDA fibroblasts

**Fig. 4:**
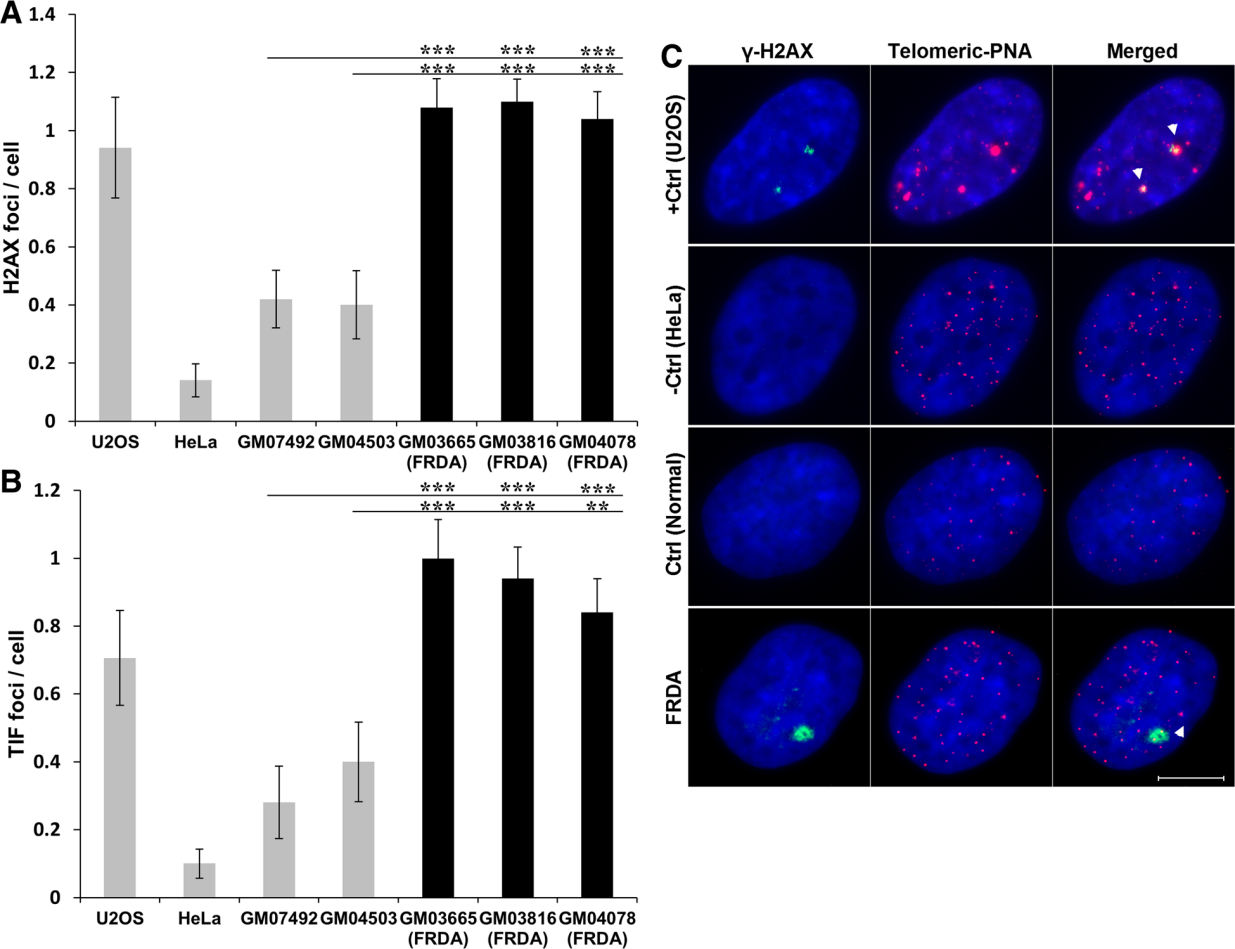
Frequencies of γ-H2AX positive foci and TIFs in FRDA fibroblast cells. **a** Average number of γ-H2AX positive foci in FRDA fibroblasts (GM03665, GM03816, GM04078), unaffected fibroblasts (GM07492, GM04503), U2OS ALT-positive control cells (+Ctrl) and HeLa ALT-negative control cells (−Ctrl), *n* = 50–100. **b** Average TIFs in each corresponding cell line, *n* = 50–100. Data are shown as mean ± SEM (^**^ *P* < 0.01 and ^***^ *P* < 0.001, evaluated by Student’s *t* test). **c** Representative images showing detection of γ-H2AX by immuno-FISH in FRDA and control (normal) fibroblast cells, U2OS ALT-positive control cells (+Ctrl) and HeLa ALT-negative control cells (−Ctrl). Interphase cells were stained with antiphospho-histone γ-H2AX (green) and telomeric Cy3-labeled PNA probes (red). Nuclei were counterstained with DAPI (blue). White arrowheads in merged images indicate TIFs (yellow). Scale bar = 10 μm. All experiments were repeated independently, at least twice

To confirm the presence of a telomere dysfunction phenotype in FRDA fibroblasts, the telomere dysfunction-induced foci (TIF) assay was performed using an antibody against DNA damage marker γ-H2AX together with the synthetic PNA telomere probe. The presence of TIFs in a cell is indicative of DNA damage at telomeres [15] and is used to quantitatively measure levels of telomeric dysfunction. As evident in Figs. 4 a and c, the frequency of γ-H2AX foci was significantly higher in FRDA cells compared to controls (*P* < 0.001). Similarly, FRDA cells had greater TIF frequencies in comparison to controls (*P* < 0.001) (Fig. 4b,c), indicating that FRDA fibroblasts have greater induced telomere dysfunction.

## Discussion

FRDA cells are known to exhibit increased susceptibility to oxidative stress, an important modulator of telomere length [4, 16]. Therefore, we hypothesised that telomeres might be shortened in FRDA patient cells. However, our initial results showed that both human and mouse FRDA fibroblasts appear to have significantly longer, rather than shorter, telomere lengths compared to normal control fibroblasts, with the caveats that there is variability within these systems and our results are based on the analysis of only three human and two mouse FRDA cell lines, which have relatively low GAA repeat numbers. This contrasts with the results of a recent study by Castaldo and colleagues that showed significant telomere shortening in human FRDA leukocytes [5]. Therefore, we further examined telomere length in our own collection of FRDA leukocyte genomic DNA samples to investigate this apparent discrepancy. However, we identified a significantly shortened mean telomere length in FRDA leukocytes compared with controls, which is in good agreement with the previous study by Castaldo and colleagues [5]. In addition, we showed that the telomere lengths from FRDA cerebellum autopsy tissues were significantly shorter than those of unaffected age-matched controls. However, we identified considerable variation in the telomere lengths of different FRDA patient leukocyte and cerebellum tissue samples and there were no correlations between telomere lengths and age of onset or GAA repeat size, which casts some doubt upon the potential use of telomere length as an effective biomarker of FRDA disease severity. However, telomere length may still be a useful biomarker of FRDA disease progression, as proposed by Castaldo and colleagues [5]. By analyzing fibroblasts, leukocytes and cerebellar tissues, we have shown that there is variation of telomere length within different FRDA cell and tissue types. This may be due to several factors, including differences in cellular replication status and different cellular environments, as the replicating fibroblasts were grown *in vitro*, while the primarily non-replicating leukocytes and cerebellar tissues were obtained *in vivo*.

We further showed that FRDA fibroblasts do not maintain telomeres due to telomerase activity, but rather due to activation of an ALT-like mechanism. The causes of ALT activation are not fully understood. However, mutations in the ATRX/DAXX chromatin remodelling complex and histone H3.3 have been found to correlate with ALT [17]. Also, it has been observed that mismatch repair (MMR) defects can facilitate ALT engagement in yeast [18]. It has also been shown that FRDA cells are more sensitive to ionising radiation than the control cells [1]. Furthermore, gene expression analysis of FRDA patient blood samples has identified reduced expression of DNA repair genes [2, 3], suggesting that FRDA may be a DNA repair-deficient disorder. This notion is supported by our current findings of high frequencies of γ-H2AX and TIFs in FRDA fibroblasts compared to the controls, indicating a higher degree of DNA damage in FRDA cells. Therefore, an ALT-like mechanism may become activated in FRDA cells due to a combination of oxidative DNA damage and defective DNA repair mechanisms. However, we also showed that FRDA fibroblasts were unable to become immortalised during long-term growth in culture, indicating that the ALT-like activity was not sufficient to overcome cellular senescence. The reason why we did not observe a complete engagement of the ALT-like pathway in FRDA telomere maintenance may be due to defective DNA repair systems or alteration of the heterochromatic state of telomeres [9]. Nevertheless, FRDA cells were found to senesce approximately 11 population doublings later than the controls. This extension of the transition time from proliferation to senescence could be partly due to the difference in the original telomere lengths of these cell lines, in addition to the selective activation of the ALT-like mechanism. We also identified a faster telomere attrition rate in FRDA cells compared with controls, suggesting telomere dysfunction, which again could be due to oxidative DNA damage and defective DNA repair mechanisms [4] or due to an altered heterochromatic state of the telomeres, resulting in the repression of telomeric recombination [9]. Accelerated loss of telomere lengths has also been reported in other neurodegenerative diseases that are associated with oxidative stress, such as Alzheimer’s disease and Parkinson’s disease, suggesting the existence of common molecular disease mechanisms [19, 20]. However, it is possible that the accelerated telomere shortening that we identified in FRDA fibroblast cells could be an artefact of *in vitro* culture and in fact *in vivo* skin cells from FRDA patients have a normal rate of telomere shortening. Therefore, the exact mechanisms of telomere shortening under conditions of oxidative stress require further investigation, for example by repeated skin biopsy sampling throughout age from individual FRDA patients or repeated tissue sampling FRDA mouse models.

Dysfunctional telomeres in FRDA cells may occur due to defects in a variety of potential genes or proteins that are essential for DNA repair and telomere maintenance, including *DNA-PKcs*, *Ku70/80*, *TRF2*, *ATM*, *PARP*, *BRCA1*, *BRCA2* and DNA helicases [21–29]. These may also include enzymes that require iron sulfur cluster (ISC) cofactors for activity. For example, recent findings indicate that RTEL1, the ISC-containing DNA helicase regulator of telomere length 1, is essential for telomere homeostasis by catalysing T-loop disassembly during S phase and therefore its deficiency is associated with removal of the T loop structures and rapid telomere shortening [30, 31]. In addition, methyl methanesulfonate sensitive 19 (MMS19), a yeast member of the cytosolic ISC assembly (CIA) machinery, has been found to function as the most interactive partner of RTEL1 [32]. Therefore, the reported sensitivity of MMS19 deficient cells to DNA damage and the presence of extended telomeres may be attributed to the function of MMS19 in telomere maintenance.

Other studies have also found a link between epigenetic status of subtelomeres and telomere length regulation, suggesting a role for subtelomeric methylation in telomere-length homeostasis [33, 34]. Telomeres have a closed conformation, mediated by the interaction of DNA with epigenetic markers. As telomeres become shorter, the heterochromatic markers are decreased from telomeres and subtelomeres, which lead to a less dense conformation, allowing a greater accessibility for telomere elongating activities [35]. An increase in the hypermethylation of the shortest telomeres has been reported in patients with Alzheimer’s disease [36], whereas, hypomethylated subtelomeres have been reported to be associated with increased telomeric shortening in patients with Parkinson’s disease [37]. It has also been shown that short telomeres with hypomethylated subtelomeres tend to be lost faster than those with enhanced subtelomeric methylation [37–40]. These results suggested that oxidative stress in neurodegenerative disorders might affect the access of the DNA methyltransferase to subtelomeres, thus resulting in subtelomeric hypomethylation and consequential progress to cell death [41]. To date, none of these telomere length-associated changes have been investigated in FRDA patients. Therefore, further analysis is required to clarify the correlation between telomere length and methylation status in FRDA cells.

## Conclusions

Overall, the results presented in this study demonstrate a telomere dysfunction phenotype and accelerated telomere shortening in FRDA fibroblasts, together with comparatively reduced telomere lengths in both FRDA leukocytes and cerebellar tissues. In addition, we observed that ALT-like inter-telomeric recombination was initiated in FRDA fibroblasts, but this was incapable of preventing accelerated telomere shortening. Our studies support an FRDA molecular disease model whereby oxidative DNA damage, in combination with defective DNA repair responses and perhaps epigenetic changes, induce suboptimal ALT-like telomeric recombination. However, it remains unclear why there is not a prolonged effect of the ALT-like mechanism on FRDA telomere length and this requires further analysis. Cell type-specific factors are likely to contribute to the telomere length maintenance, hence further experiments using different cell types will further enhance our understanding of the correlation between telomere length in FRDA with various genetic and cellular risk factors. Furthermore, our observations may have an impact on future FRDA therapeutic strategies. Since telomere shortening plays an important role in human cell viability, telomere length may yet prove to be a useful biomarker of cumulative exposure to oxidative stress and defective DNA responses in FRDA disease progression and subsequent amelioration by therapy.

## Methods

### Cell culture

Mouse fibroblast cell lines were established from the kidney tissues of C57BL/6 J (B6) mice and previously reported *FXN* YAC transgenic mouse models: Y47R (9 GAA repeats), YG8R (90 and 190 GAA repeats) and YG22R (190 GAA repeats) [42, 43]. All human fibroblasts derived from FRDA patients and unaffected controls were obtained from the Coriell Cell Repository (Table 1). Cells were cultured in DMEM culture medium (Gibco) supplemented with 10-15 % fetal bovine serum (Gibco) and 2 % penicillin-streptomycin (Gibco) at 37 °C with 5 % CO_2_. Mouse lymphoma LY-R (radio-resistant) and LY-S (radio-sensitive) cells were cultured in RPMI 1640 medium (Invitrogen) supplemented with 10 % FBS and 2 % Pen-Strep at 37 °C with 5 % CO2. U2OS (human osteosarcoma) and HeLa (human cervical carcinoma) cell lines were purchased from ECCC (European Collection of Cell Culture) and ATCC (American Tissue Culture Collection), respectively. The U2OS cell line was grown in McCoy’s culture medium (Sigma-Aldrich) supplemented with 10 % FBS, 2 % Pen-Strep and 2 mM glutamine (Gibco) at 37 °C with 10 % CO_2_. The HeLa cell line was grown in DMEM culture medium supplemented with 10 % FBS and 2 % Pen-Strep at 37 °C with 10 % CO_2_. For growth curve analysis, FRDA and control fibroblast cell lines were serially passaged by re-seeding at 100,000 cells/25 cm^2^ flasks (Fisher Scientific) and harvesting at 80 % confluence from earliest passage until they entered replicative senescence. For each harvest, cells were counted with a haemocytometer (Invitrogen) and transferred every 6–7 days. Cell population doublings per passage (PDP) and cumulative population doublings (CPD) were calculated from the first passage onward by the following formula [44]: PD = (Log N_1_ /Log2)-(Log N_o_ / Log2); CPD_n_ = ΣPD_(1, n)_, where N_o_ is the number of cells at the beginning; N_1_ is the number of cells at the end of each cell culture period and n is the number of passages. β-galactosidase activity was determined as described [45].

### DNA extraction and telomere length measurement

Genomic DNA was extracted from FRDA and control human and mouse fibroblasts, blood samples and cerebellum autopsy tissues by standard phenol/chloroform extraction and ethanol precipitation. Telomere length was determined by a previously described quantitative polymerase chain reaction (qPCR) method [46]. In brief, the relative telomere length was calculated from the ratio of the telomere (T) repeat copy number to a single gene (36B4, which encodes the acid ribosomal phosphoprotein PO) (S) copy number (T/S ratio) for each sample using standard curves. For quality control, all qPCR telomere length measurements were performed in triplicate. To quantify telomere shortening per PD, the following formula was used: (N_1_-N_o_) / CPD, where N_o_ is the telomere length at the initial passage and N_1_ is the telomere length at the later passage.

### Cytogenetic analysis

Interphase quantitative fluorescent in situ hybridisation (IQ-FISH) was performed using the FITC-conjugated peptide nucleic acid (PNA) telomeric oligonucleotide (CCCTAA)_3_ probe as previously described [47]. Images of interphase cells were acquired on a digital fluorescence microscope (Zeiss Axioskop 2) equipped with a CCD camera (Photometrics) and Smart Capture software (Digital Scientific) using fixed exposure time of 0.5 second and magnification of 63×. Telomere fluorescence intensity per cell was analysed using IP Lab software (Digital Scientific) and the average signal was evaluated by subtracting the background signal from the total telomeric signal intensity.

Chromosome orientation fluorescence in situ hybridisation (CO-FISH) was performed as previously described [48]. Briefly, cells were grown in the presence of BrdU/BrdC (3:1) (Sigma Aldrich) at a concentration of 1 × 10^−5^ for 24 h. Slides containing chromosome preparation were stained with DNA-binding fluorescent dye Hoechst 33258 (0.5 μg/ml; Sigma Aldrich) for 15 min at room temperature and were exposed to 365 nm UV light (Stratelinker 1800 UV irradiator) for 30 min. The nicked BrdU-substituted DNA strands were then digested by 3U/ml of Exonuclease III (Promega) in the buffer supplied by the manufacturer at room temperature for 10 min. *In situ* hybridisation was performed using the Cy3-conjugated PNA telomeric oligonucleotide (CCCTAA)_3_ probe as previously reported [49]. Images of metaphase spreads were acquired using a Zeiss fluorescence microscope equipped with a CCD camera (Photometrics) and ISIS Capture software (*in situ* imaging system “ISIS”, Meta Systems, Altlussheim, Germany).

Telomere dysfunction induced foci (TIF) assays were performed as previously described [50]. Briefly, cells were grown on poly-L-lysine-coated slides (Poly-Prep slides, Sigma-Aldrich) for 24 h prior to immunofluorescence staining. The slides were fixed in 4 % formaldehyde in PBS for 15 min and were permeabilised with 0.2 % Triton X-100 (Sigma-Aldrich) for 10 min at 4 °C. The cells were then blocked with 0.5 % BSA in PBS for 30 min. 100 μl of mouse monoclonal antiphospho-histone H2AX (Ser 139, Millipore) was added to the slides at the desired concentration (1:500 in 0.5 % BSA/PBS) and the slides were incubated for 1 h in a humid container. After three washes in TBST (Tris-Buffered Saline with 0.1 % Tween20) for 5 min, the slides were incubated with 100 μl of secondary FITC-conjugated anti-mouse IgG antibody (Sigma) at the desired concentration (1:1000 in 0.5 % BSA/PBS) for 1 h in a humid container. The slides were washed again as above and hybridised for 2 min at 70-75 °C with Cy3-conjugated PNA (CCCTAA)_3_ probe. The slides were analysed using a Zeiss fluorescence Axioplan microscope equipped with a CCD camera and Smart Capture software. The frequency of γH2AX was analysed from the TIF results since the TIF protocol simultaneously detects γH2AX foci and telomeres.

For immuno-FISH, cells were grown on poly-L-lysine-coated slides (Poly-Prep slides, Sigma-Aldrich) for 24 h, then fixed in ice-cold methanol-acetone (1:1) for 10 min and washed three times in PBS. The cells were then blocked with 1 % NCS (Newborn Calf serum) in PBS for 30 min. 75 μl of promyelocytic leukemia (PML) primary antibody (Rabbit polyclonal antibody against PML, ab53773, Abcam) was added to the slides at the desired concentration (1:100 in 1 % NCS/PBS) and the slides were then incubated for 1 h in a humid container. The slides were then washed three times in PBS for 5 min. 75 μl of anti-rabbit secondary antibody labelled with FITC (F1262-1ML, Sigma) was added to the slides at the desired concentration (1:100 in 1 % NCS/PBS) and the slides were subsequently incubated for 1 h in a humid container. After rinsing in PBS, the FISH method was applied with a Cy3-conjugated PNA probe (CCCTAA)_3_ followed by standard formamide and SSC washes. Cells were counterstained with DAPI and were examined on a Zeiss Axioplan fluorescence microscope equipped with a CCD camera (Photometrics) and Smart Capture software (Digital Scientific) using fixed exposure time of 1 s and magnification of 1000×. The co-localisation of PML and telomere signals was analysed using ImageJ software (National Institutes of Health, Bethesda, MD).

### Telomerase assay

Cells (10^5^-10^6^) were lysed in 200 μl of CHAPS buffer, containing RNase inhibitor, and were incubated at 4 °C for 30 min. The lysates were then centrifuged at 15700 X g for 20 min at 4 °C. The protein concentration was measured using a Pierce® BCA Protein Assay Kit (Thermo scientific) following the manufacturer’s instructions. In order to obtain the final concentration of 125 ng/μl, the protein concentration of the tested samples was measured from the standard curve and the samples were diluted in CHAPS lysis buffer accordingly. Telomerase activity was measured using TRAPEZE® Telomerase Detection Kit (Chemicon, Millipore) according to the manufacturer’s instructions.

### Gene expression of hTERT using qRT-PCR

Total RNA was extracted from 10^6^ cells using the Trizol® method (Invitrogen) and reverse transcribed using AMV reverse transcriptase (Invitrogen) with random hexanucleotide primers following the manufacturer’s instructions. hTERT mRNA expression levels were quantified by qRT-PCR using an ABI Prism 7900HT Sequence Detection System and TaqMan® Fast Universal Master Mix (Applied Biosystems) [51]. An hTERT assay (Applied Biosystems), containing hTERT forward (5′-GAGCTGACGTGGAAGATGAGC-3′) and reverse (5′-GGTGAACCTCGTAAGTTTATGCAA-3′) primers and a TaqMan TAMRA™ probe (5′-CACGGTGATCTCTGCCTCTGCTCTCC-3′) giving a 260 bp fragment, and a GAPDH assay (Assay ID: Hs99999905_m1, 124 bp amplicon size, Applied Biosystems), containing GAPDH forward and reverse primers and a TaqMan MGB probe, were used in this experiment. The cycling protocol consisted of 20 s at 95 °C, followed by 50 cycles (95 °C for 1 s and 60 °C for 20 s). Reactions were carried out in triplicate for each sample.

### Statistical analysis

Comparisons between population doublings were assessed by two-way analysis of variance (ANOVA) while relationships between telomere length and other variables were calculated by multiple regression analysis. All other data were analysed by the Student’s *t* test, with a significance value set at *P* < 0.05.

## Additional files

Additional file 1: Figure S1. Variation in telomere length of human leukocytes. Telomere length measurements using qPCR showed that there was a variation in the telomere length of the FRDA patients and control leukocytes. Data are shown as mean ± SEM. QPCR runs were performed in triplicate and repeated twice from independent samples. Additional file 2: Figure S2. Effect of gender and size of GAA repeats on telomere length in FRDA leukocytes. (A) Mean telomere length values ± SEM of FRDA male and female leukocytes (*P* = 0.4, evaluated by Student’s *t* test). (B and C) Linear regression analysis was used to assess the correlation between telomere length and size of the smaller (B) or larger GAA repeats (C) in FRDA leukocytes. Additional file 3: Table 1. FRDA and control DNA samples from human cerebellum tissues. Additional file 4: Figure S4. Telomere length analysis in FRDA cerebellum tissues. (A) Box-plot display of telomere length distributions in the cerebellum tissues of FRDA patients and age-matched controls (^*^ *P* < 0.05, evaluated by Student’s *t* test). QPCR runs were repeated twice from independent samples. (B, C and D) Linear regression analysis was used to assess the correlation between telomere length and age of onset (B), size of the larger (C) or smaller GAA repeats (D) in FRDA cerebellum tissues, *n* =10-13. Additional file 5: Figure S5. Telomerase activity in FRDA fibroblasts. (A) Telomerase activity determined by the TRAP assay in FRDA cells. The breast cancer cell line 21NT was used as the positive control. No template control (NTC) and minus telomerase control (MTC) served as the negative controls. Data are shown as mean ± SEM. The experiment was repeated twice from independent samples. (B) Products of hTERT transcripts in FRDA fibroblast cells. The breast cancer cell lines 21NT and 21MT were used as the positive controls. Normal human fibroblast GM07492 and GM08399 cell lines, normal breast epithelial cell line and NTC (No Template Control) served as the negative controls. Additional file 6: Figure S6. Size analysis of associated and non-associated PML bodies with telomeres. The sizes of the PML bodies (associated and non-associated) were analysed in FRDA cell lines (*P* = 0.44) and positive control U2OS (*P* = 0.99, evaluated by Student’s *t* test) using ImageJ software. Data are shown as mean ± SEM, *n* = 50–150. Additional file 7: Figure S7. β-galactosidase detection in FRDA Fibroblasts. Early and late passaged FRDA fibroblast cells were assayed for β-galactosidase activity. Growing 293 T cells and senescing human mammary epithelial cells (HMECs) were used as negative (−ve) and positive (+ve) controls respectively, scale bar = 100 μm.

## Abbreviations

FRDA: Friedreich Ataxia
TL: Telomere length
ALT: Alternative lengthening of telomeres
T-SCE: Telomeric sister chromatid exchanges
PML: Promyelocytic leukemia protein
CPD: Cumulative population doublings
IQ-FISH: Interphase quantitative fluorescent in situ hybridization
CO-FISH: Chromosome orientation fluorescence in situ hybridisation
TIF: Telomere dysfunction induced foci
hTERT: Human telomerase reverse transcriptase
