# Supplementary figures and images for: Identification of telomere dysfunction in Friedreich ataxia
Source: Mol Neurodegener. 2015 Jun 10;10:22. doi: 10.1186/s13024-015-0019-6 (PMC4462004; doi:10.1186/s13024-015-0019-6)

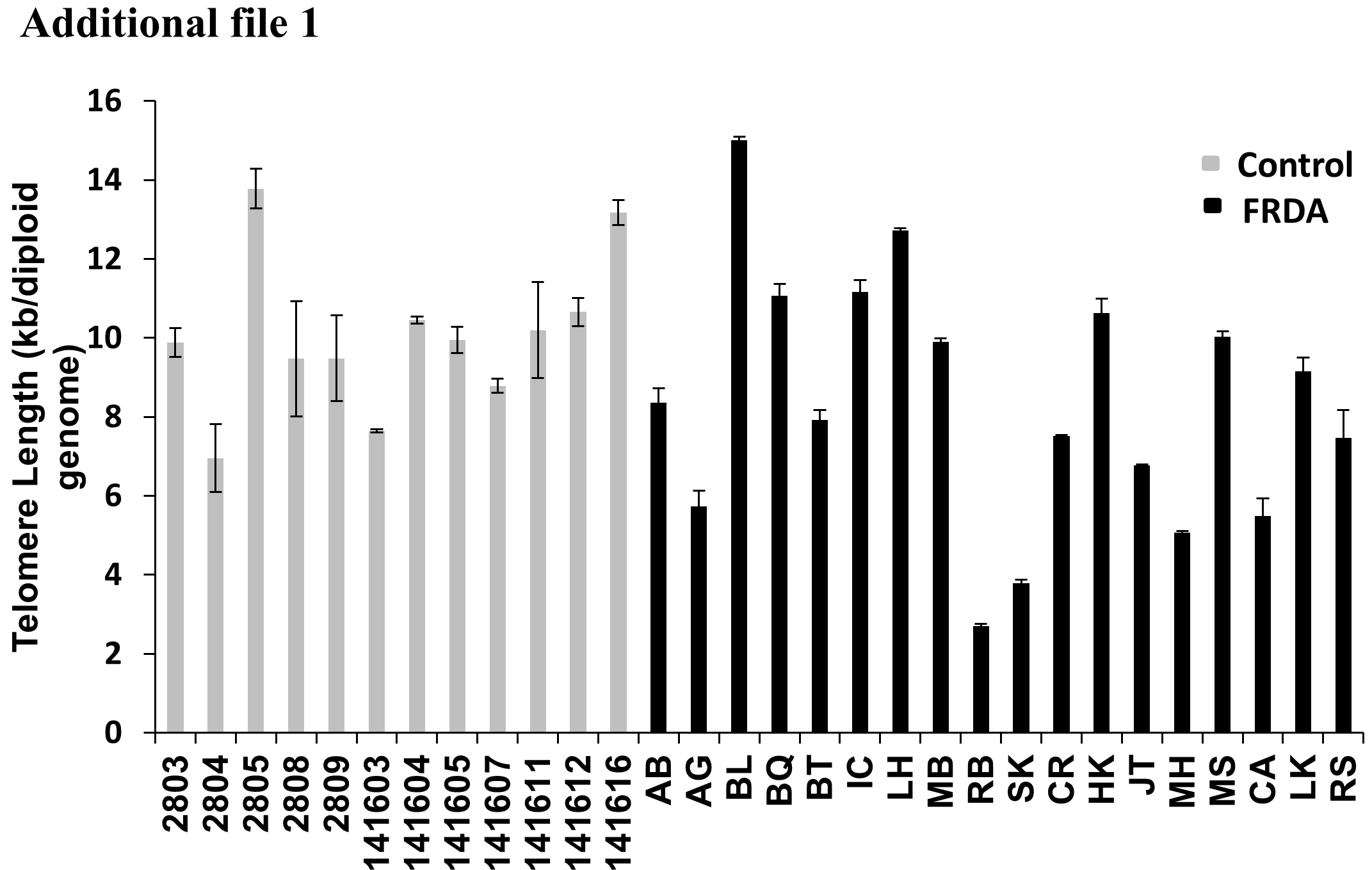

Supplement: Additional file 1: Figure S1. — Variation in telomere length of human leukocytes. Telomere length measurements using qPCR showed that there was a variation in the telomere length of the FRDA patients and control leukocytes. Data are shown as mean ± SEM. QPCR runs were performed in triplicate and repeated twice from independent samples. [file 13024_2015_19_MOESM1_ESM.tiff]

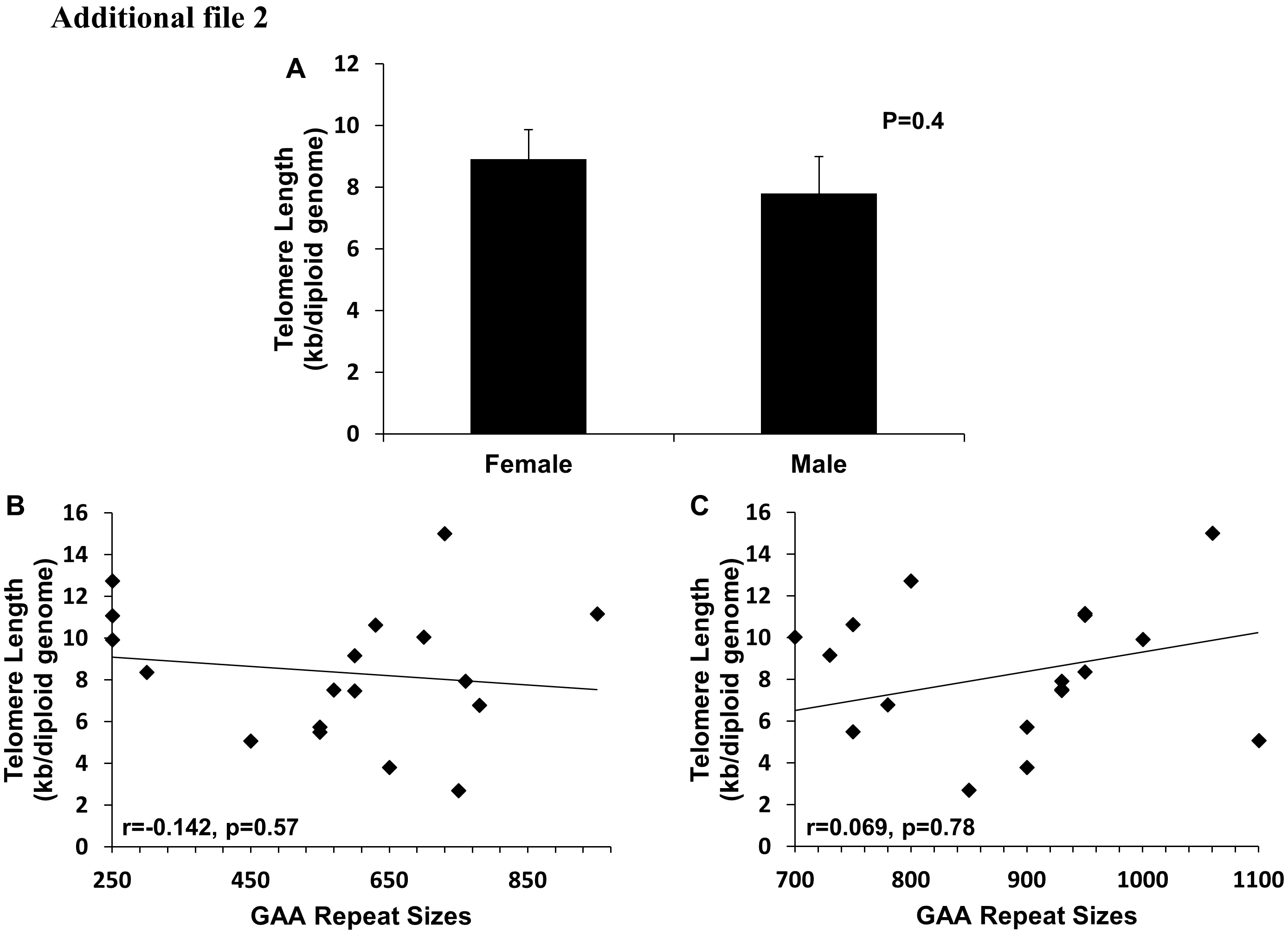

Supplement: Additional file 2: Figure S2. — Effect of gender and size of GAA repeats on telomere length in FRDA leukocytes. (A) Mean telomere length values ± SEM of FRDA male and female leukocytes (P = 0.4, evaluated by Student’s t test). (B and C) Linear regression analysis was used to assess the correlation between telomere length and size of the smaller (B) or larger GAA repeats (C) in FRDA leukocytes. [file 13024_2015_19_MOESM2_ESM.tiff]

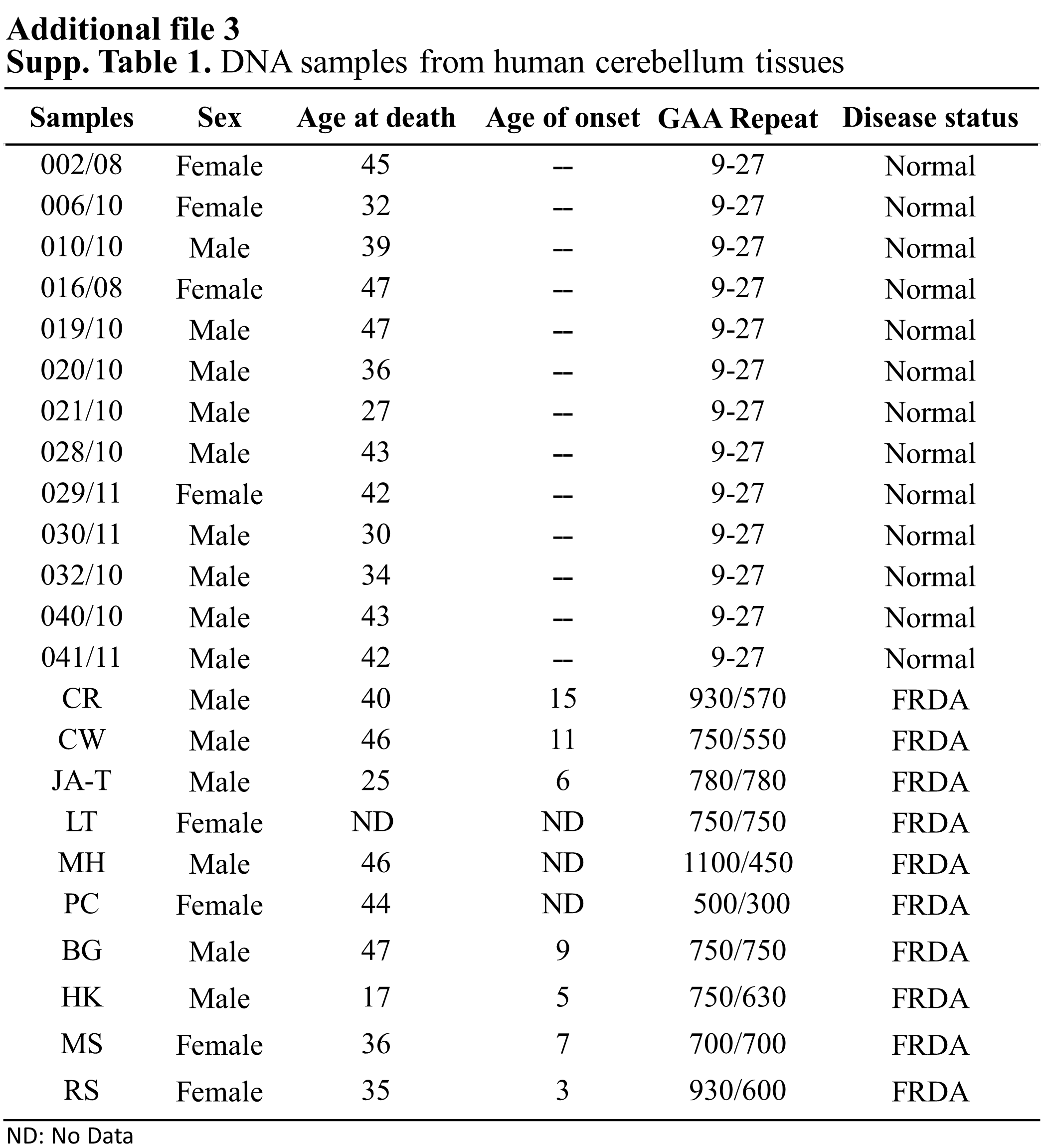

Supplement: Additional file 3: Table 1. — FRDA and control DNA samples from human cerebellum tissues. [file 13024_2015_19_MOESM3_ESM.tiff]

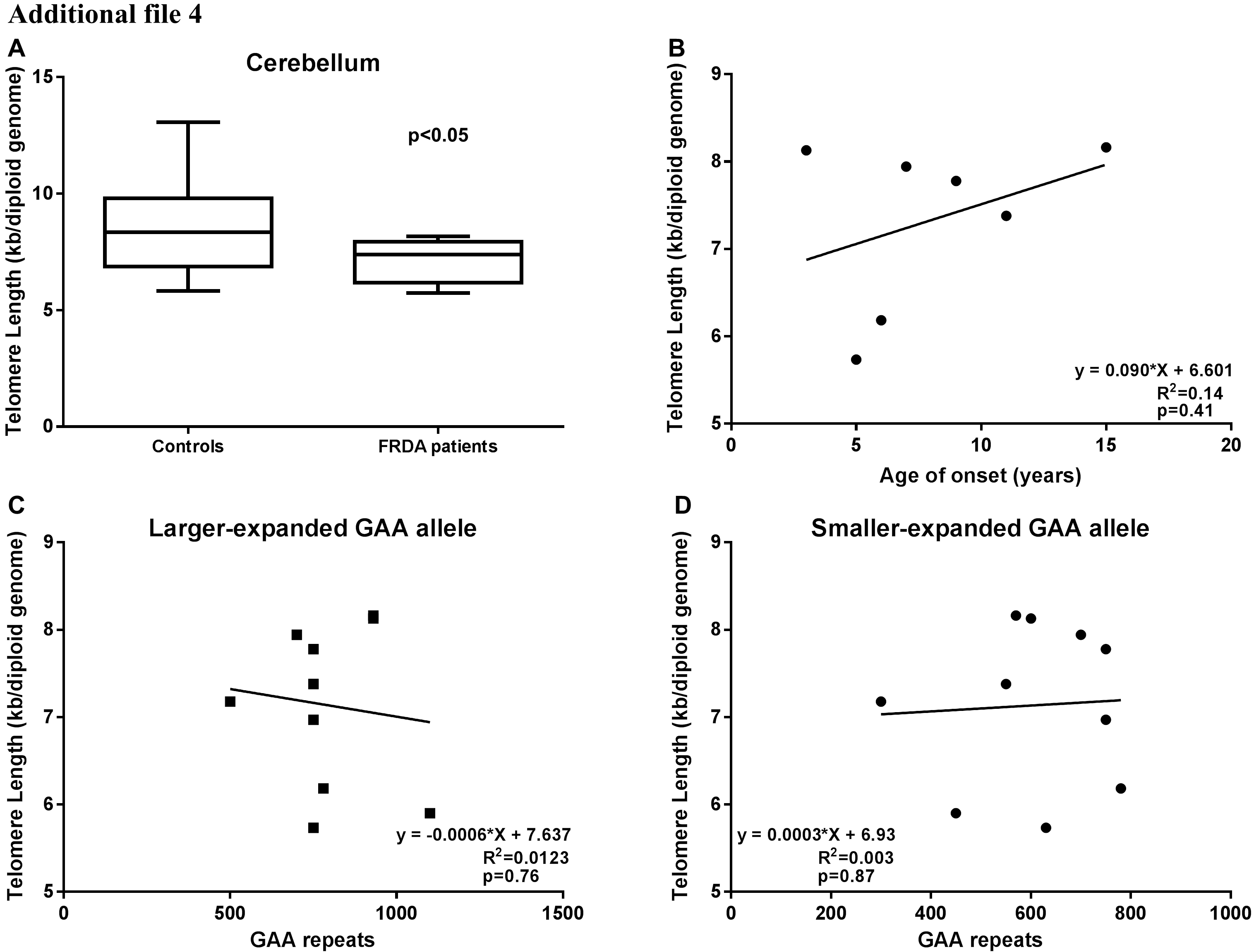

Supplement: Additional file 4: Figure S4. — Telomere length analysis in FRDA cerebellum tissues. (A) Box-plot display of telomere length distributions in the cerebellum tissues of FRDA patients and age-matched controls (* P < 0.05, evaluated by Student’s t test). QPCR runs were repeated twice from independent samples. (B, C and D) Linear regression analysis was used to assess the correlation between telomere length and age of onset (B), size of the larger (C) or smaller GAA repeats (D) in FRDA cerebellum tissues, n =10-13. [file 13024_2015_19_MOESM4_ESM.tiff]

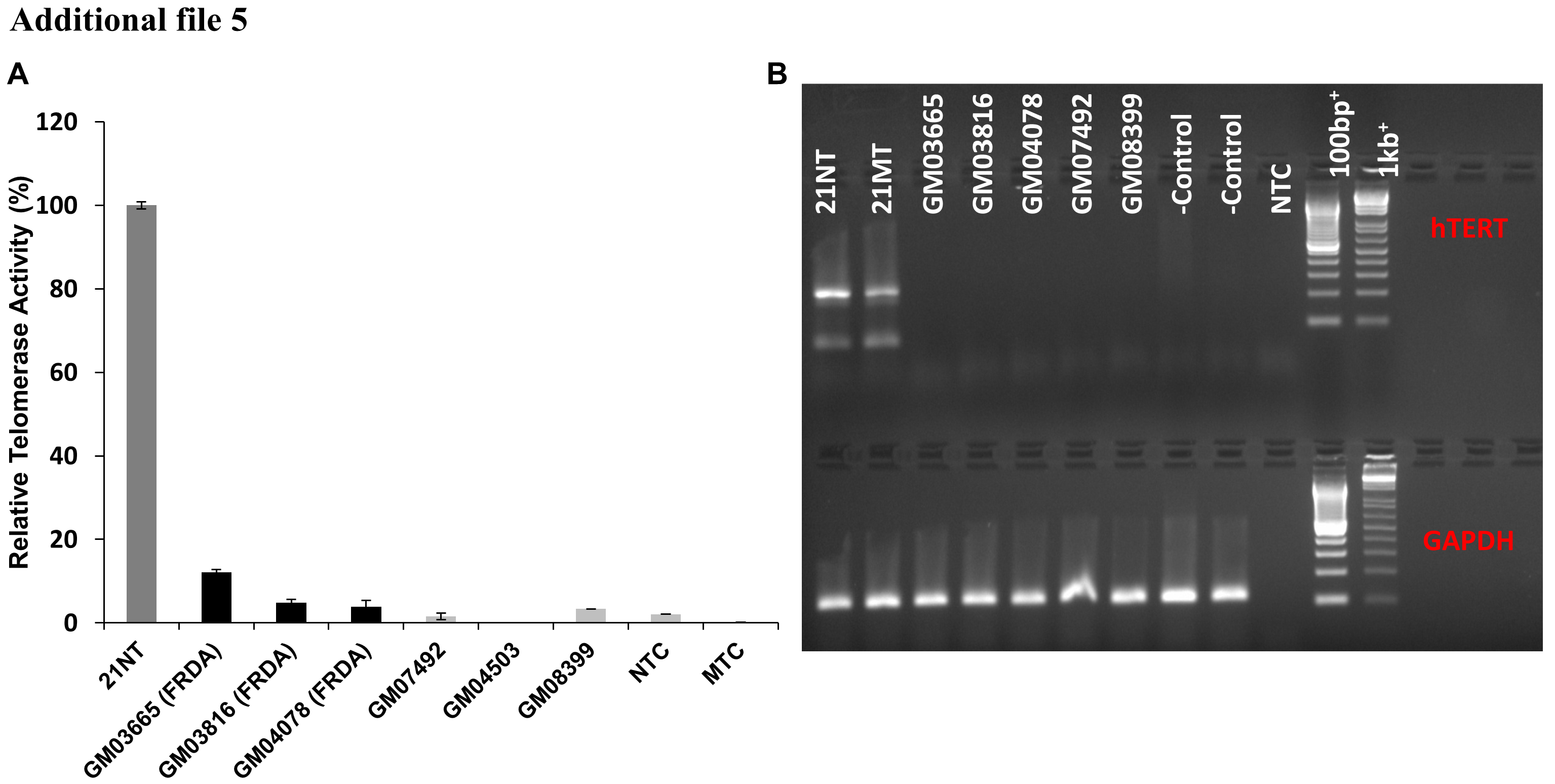

Supplement: Additional file 5: Figure S5. — Telomerase activity in FRDA fibroblasts. (A) Telomerase activity determined by the TRAP assay in FRDA cells. The breast cancer cell line 21NT was used as the positive control. No template control (NTC) and minus telomerase control (MTC) served as the negative controls. Data are shown as mean ± SEM. The experiment was repeated twice from independent samples. (B) Products of hTERT transcripts in FRDA fibroblast cells. The breast cancer cell lines 21NT and 21MT were used as the positive controls. Normal human fibroblast GM07492 and GM08399 cell lines, normal breast epithelial cell line and NTC (No Template Control) served as the negative controls. [file 13024_2015_19_MOESM5_ESM.tiff]

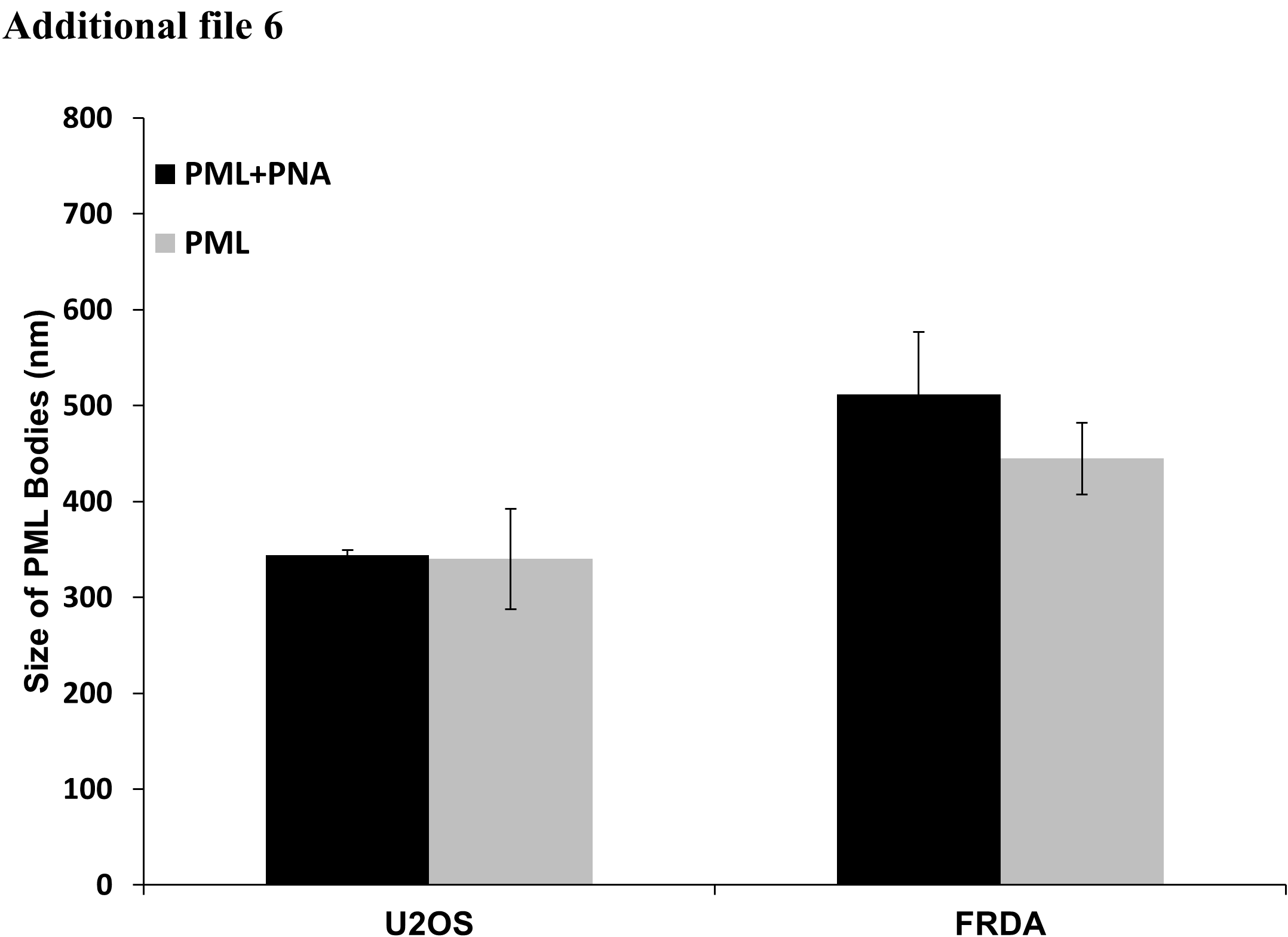

Supplement: Additional file 6: Figure S6. — Size analysis of associated and non-associated PML bodies with telomeres. The sizes of the PML bodies (associated and non-associated) were analysed in FRDA cell lines (P = 0.44) and positive control U2OS (P = 0.99, evaluated by Student’s t test) using ImageJ software. Data are shown as mean ± SEM, n = 50–150. [file 13024_2015_19_MOESM6_ESM.tiff]

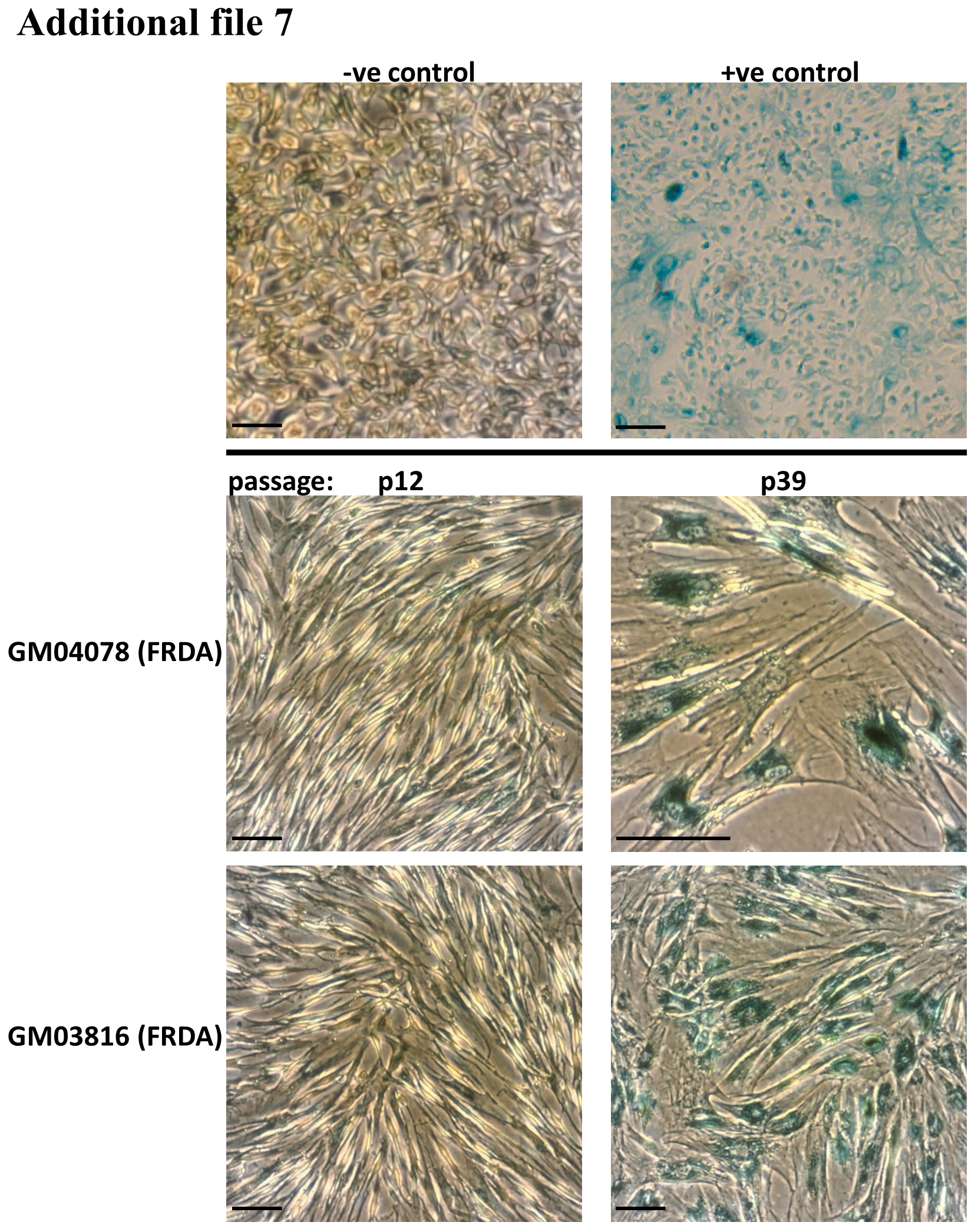

Supplement: Additional file 7: Figure S7. — β-galactosidase detection in FRDA Fibroblasts. Early and late passaged FRDA fibroblast cells were assayed for β-galactosidase activity. Growing 293 T cells and senescing human mammary epithelial cells (HMECs) were used as negative (−ve) and positive (+ve) controls respectively, scale bar = 100 μm. [file 13024_2015_19_MOESM7_ESM.tiff]
